# Effectiveness of Information and Communication Technology (ICT) Interventions in Elderly’s Sleep Disturbances: A Systematic Review and Meta-Analysis

**DOI:** 10.3390/s21186003

**Published:** 2021-09-08

**Authors:** Seonheui Lee, Soyoung Yu

**Affiliations:** 1 Department of Nursing Science, College of Nursing, Gachon University, Incheon 21936, Korea; sunarea87@gachon.ac.kr; 2 College of Nursing, CHA University, Pocheon 11160, Gyeonggido, Korea

**Keywords:** aged, information and communication technologies (ICT), meta-analysis, sleep-wake disorders, systematic review

## Abstract

Sleep is a crucial factor for human health and is closely related to quality of life. Sleep disturbances constitute a health problem that should be solved, especially when it affects the elderly. This study aims to examine the effectiveness of information and communication technologies (ICT) interventions in managing sleep disturbances in the elderly. The study used a systematic review of three databases: Ovid-Medline, Ovid-EMBASE, and the Cochrane library database for papers published till 15 April 2021. Two authors independently selected and screened relevant studies based on predefined inclusion criteria. The meta-analysis of randomized controlled trials (RCTs) was carried out using Review Manager 5.4. Two authors independently screened the titles and abstracts of 4297 studies considering both inclusion and exclusion criteria. The complete texts of 47 articles were then evaluated, 31 articles were excluded, and finally, 16 articles were selected. Our meta-analysis showed that the cognitive-behavioral therapy for insomnia (CBT-I) group had a significantly reduced Insomnia Severity Index (ISI) compared to the control group (−4.81 [−5.56, −4.06], *p* < 0.00001, I^2^ = 83%) in RCTs, with a significant reduction in ISI (3.47 [1.58, 5.35], *p* = 0.0003) found in quasi-experimental studies. A significant improvement was found in total sleep time in the CBT-I group compared to the control group (29.24 [15.41, 43.07], *p* <0.0001) in RCTs, while the CBT-I group showed significantly reduced wake time after sleep onset compared to the control group (−20.50 [−26.60, −14.41], *p* < 0.00001). In addition, a significant reduction in depression was found in the CBT-I group compared to the control group (−2.11 [−2.85, −1.37], *p* < 0.00001, I^2^ = 0%) in RCTs. The quality of life–mental component score (5.75 [1.64, 9.87], *p* = 0.006, I^2^ = 0%) and the quality of life–physical component score (5.19 [0.76, 9.62], *p* = 0.02, I^2^ = 0%) showed significant improvement in the CBT-I group compared to the control group. ICT interventions showed positive effects on sleep disturbances of the elderly, specifically confirming the positive effect on depression and quality of life as well as the indicators directly related to sleep such as ISI and quality of sleep. Thus, the application of ICT in the healthcare sector will be greater in the future, with changes in the nursing education and practice guidelines so that nurses can play a pivotal role in promoting health behaviors such as sleep-related quality of life and daily activities of the elderly.

## 1. Background

One of the important factors affecting daily human life concerns sleep-related issues such as sufficient sleep time and quality of sleep. Sleep disturbances occur for various reasons and, besides affecting an individual’s life, might also lead to much expenditure on diagnosis and treatment [1]. Therefore, an appropriate level of sleep, both quantitatively and qualitatively, is essential for optimal physical and mental well-being. However, lack of sleep has become a widespread and urgent health problem in today’s fast-changing society [2]. Sleep disturbances and sleep disorders are two distinct concepts, with the former occurring as a result of the latter. Sleep disturbances can be defined as certain symptoms characterized by reduced sleep duration and/or poor sleep quality [3]. Lack of sleep is associated with excessive daytime sleepiness, depressed moods, and difficulty in concentration, as well as insidious and long-term consequences such as chronic diseases. In Canada, 32% of adults (aged 18–64) sleep less than seven hours a night. Additionally, about 70 million adults in the United States and 45 million adults in Europe suffer from chronic sleep disorders [2,4,5].

Several recent studies have reported using information and communication technologies (ICT)-based interventions to solve such sleep disturbances. Some of the systematic reviews of the related literature are as follows: Cheng and Dizon [6] carried out a systematic review (including randomized controlled trials (RCTs)) and meta-analysis of works published between 1990 and 2011 to evaluate the efficacy of computerized cognitive behavioral therapy for insomnia (CCBT-I) in 18 years and older individuals. The review focused on the effects of CCBT-I on sleep quality, sleep efficiency, number of awakenings, and sleep onset latency. Considering 533 eligible studies, the authors found evidence supporting the effectiveness of CCBT-I as a self-help therapy in low-intensity treatment for insomnia. Another study on sleep considered ICT intervention through Internet-delivered CBT-I (eCBT-I). Zachariae et al. [7] carried out a systematic review and meta-analysis of experimental studies published between 1991 and 2015 to estimate the effectiveness of eCBT-I in individuals aged 18 years and older. The review primarily focused on improving insomnia severity, sleep efficiency, subjective sleep quality, wake time after sleep onset, sleep onset latency, total sleep time, and the number of awakenings during nights at post-treatment by applying eCBT-I. In 11 studies, the authors found evidence supporting the use of the Internet in CBT-I. These studies focused on sleep intervention, but the subjects were not elderly individuals.

ICT-based interventions apply to all age groups, especially the elderly. Technology use by the elderly has been increasing, following the trend of technology use by the general population. In the United States, 59% of adults aged 65 or older currently use the Internet compared to 53% in 2012. Similarly, the number of older adults in the United States who use cell phones increased from 69% in 2012 to 77% in 2014 [8], with the latest statistics showing that 80% of older adults (65 and older) presently own some type of cell phone, 47% of which are smartphones [9]. Similar trends are found in Europe, where 82% of the population use the Internet, 57% of whom are in the 55–74 age group. In Europe, 86% of older adults are reported to own mobile phones [10]. One can thus reasonably assume that a large percentage of the elderly own and use smartphones or other Internet-enabled mobile devices (67% in Europe versus 72% in the United States) [10,11].

A few studies have shown that sleep behaviors can be easily monitored and characterized through technology, with potential intervention through mobile devices such as smartphones and tablets [12,13]. These studies focus on the elderly, but rather than focusing on sleep, they deal with general content such as physical activity and lifestyle. Therefore, in this study, authors conducted a systematic review and meta-analysis to determine the effectiveness of ICT interventions in sleep disturbances of the elderly using the Preferred Reporting Items for Systematic Reviews and Meta-Analyses (PRISMA) guidelines.

## 2. Methods

This systematic review was carried out after registering the research protocol with the Korea Health Industry Development Institute (KHIDI) prior to beginning the research. The protocol registered with KHIDI cannot be modified arbitrarily, and the pre-selected PICO or outcome indicators cannot be fixed by the researcher, preventing the selective reporting bias.

### 2.1. Search Strategy

The authors searched all the relevant studies published prior to 15 April 2021. We conducted the article search from three databases: Ovid-Medline (1946 to June 2021), Ovid-EMBASE (1974 to June 2021), and the Cochrane library. All papers published prior to the date the search was conducted were subject to analysis. To supplement the comprehensiveness of the search, additional documents were found through a manual search; their references were also searched and added.

Relevant studies were retrieved using keywords obtained from terms related to the elderly, ICT intervention, and sleep disorders.

Search terms related to the elderly are aged, older, older people, older person, older adult, elderly people, elderly, geriatric, gerontopsychiatry, senior, senium, aging, and older frail adult.

Keywords for ICT intervention are mobile application, cell phone-based, cellular phone-based, computer communication, computer-mediated, communication network, computer technology, computing technology, digital health, educational technology, e-health, ICT, information and communication technology, internet-based, mHealth, mobile phone-based, online system, smart health, smartphone-based, telecare, telecommunication, telecomputing, telemedicine, telenursing, telephone-based, web-based, smart technology, ICT-based system, telemonitoring, telephone-based, telehealth, phone-based intervention, remote consultation, sleep healthy using the Internet, social media, and audiovisual.

Terms related to sleep disorders are sleep, sleep disorder, dyssomnia, insomnia, sleepless, circadian, sleep-wake, sleep disturbance, sleep disruption, and sleep impairment. All search terms and medical subject headings (MeSH) are presented in Table S2.

### 2.2. Eligibility Criteria and Study Selection

**Figure 1:**
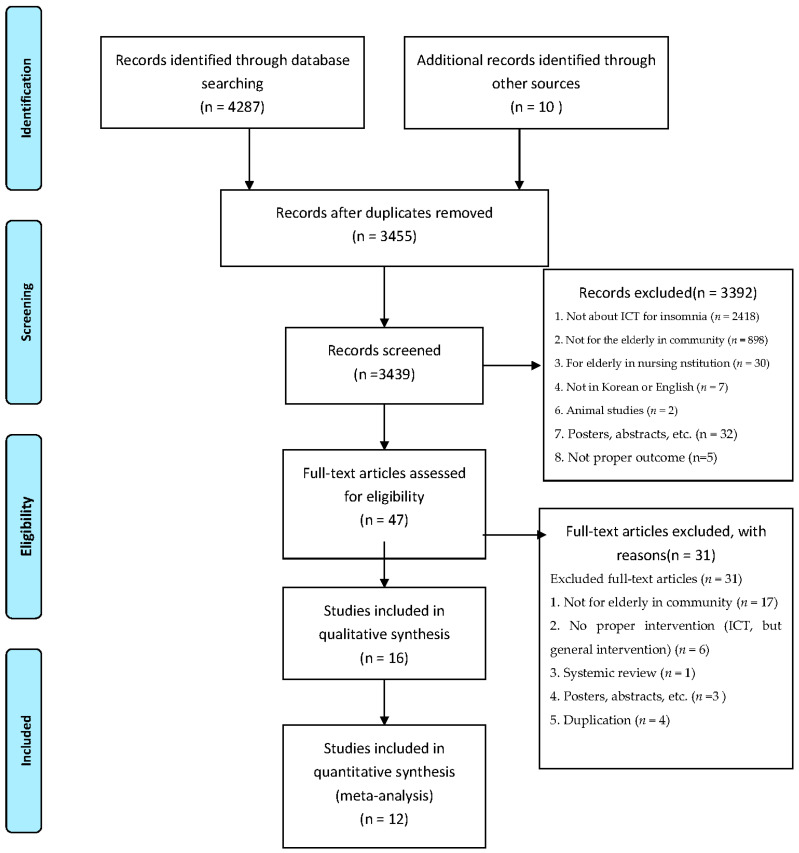
Flow chart of the study selection.

Two authors (LSH and YS) of this study selected 4297 potentially relevant studies, and they excluded 3392 of them on examining their titles and abstracts based on exclusion criteria. LSH and YS independently selected relevant studies based on certain predefined inclusion criteria: (a) studies involving the elderly aged 60 years or older, (b) studies of ICT interventions designed to manage the elderly’s insomnia, and (c) studies including protocols and reviews of latest ICT interventions. LSH and YS excluded studies meeting the following criteria: (a) posters, abstracts, animal studies, studies published that were not in English or Korean, and duplicated studies; (b) studies not drawing proper outcomes. Two reviewers independently screened the titles and abstracts based on inclusion and exclusion criteria. They examined the complete texts of relevant studies, evaluated 47 of them, and then excluded 31 of these for the following reasons: 17 included no relevant patients, 6 included no relevant interventions, 3 were gray literature, 4 were duplicated studies, and 1 was a systemic review. Finally, 16 studies were selected for analysis (see Figure 1). The authors of this study checked the reliability using Cohen’s kappa coefficient (*k* = 0.85). Disagreements regarding eligibility were resolved through discussions. Details of this are presented in the Open Science Framework (DOI 10.17605/OSF.IO/KMRUA).

### 2.3. Data Items and Collection Process

One reviewer extracted the study data independently and checked them using a data collection form predefined by mutual agreement. LSH and YS extracted some components of the population required for ICT intervention such as age, sex, and inclusion and exclusion criteria such as medical history and ISI score. LSH and YS also extracted some outcomes such as quality of sleep, the severity of insomnia, depression, quality of life, acceptance degree, satisfaction, sleeping pattern (sleeping hours, sleep stage, sleep score), physical activity level (indicators of physical activity levels, such as the following example: The Pepper Center Tool for Disability, The Patient Health Questionnaire-8), heart rate, and saturation of percutaneous oxygen (Sp02). Regarding the quality of sleep indicators (Pittsburgh Sleep Quality Index (PSQI), original version), lower scores represent good sleep quality, while higher scores indicate poor sleep quality. Sleep quality has an absolute value of 5, with a score lower than 5 indicating good sleep and a score higher than 5 indicating poor sleep. As for sleep indicators such as the severity of insomnia (Insomnia Severity Index (ISI)), higher scores indicate more severe insomnia. Scores lower than 8 indicate no clinical insomnia, 8 to 14 indicate mild clinical insomnia, 15 to 21 indicate moderate insomnia, and 22 to 28 indicate severe insomnia.

With regard to indicators such as depression (Geriatric Depression Scale (SGDS)), higher scores indicate a more depressed condition. Additionally, for indicators of quality of life (Euro Quality of Life–5 Dimension (EQ-5D)), lower scores indicate lower quality of life.

### 2.4. Risk of Bias in Individual Studies

Two authors assessed the studies for quality using the Cochrane risk of bias tool (RoB) for randomized controlled trials (RCTs). The assessment consisted of seven domains: random sequence generation (selection bias), allocation concealment (selection bias), blinding of participants and personnel (performance bias), blinding of outcome assessment (detection bias), incomplete outcome data (attrition bias), selective reporting (reporting bias), and others (other biases). Each RoB domain showed “low”, “high”, or “unclear” risk. In case of disagreement, the two reviewers discussed and decided the outcome.

### 2.5. Statistical Analysis

We used Review Manager version 5.4 software [14] and a two-tailed significance test (*p* < 0.05) for analysis. We calculated the odds ratios (ORs) and 95% confidence interval (CIs) and reported the heterogeneity between pooled studies using the Chi-squared statistic and I2. Chi-squared was considered insignificant when *p* > 0.1, and heterogeneity (I2) was rated as not important (0–40%), moderate (30–60%), substantial (50–90%), or considerable (75–100%). We used forest plots to display summary statistics.

## 3. Results

### 3.1. Characteristics of Included Studies

Table S1 gives the year of publication, country, research design, participant characteristics, and intervention methods of the 16 studies considered for analysis. By country, the United States accounted for the largest number of studies with ten, followed by Sweden with two, and South Korea, Australia, New Zealand, the Netherlands, and Taiwan with one each. As for publication year, 2019 and 2020 witnessed the highest number of papers published, with four each.

### 3.2. Quality Assessment

**Figure 2:**
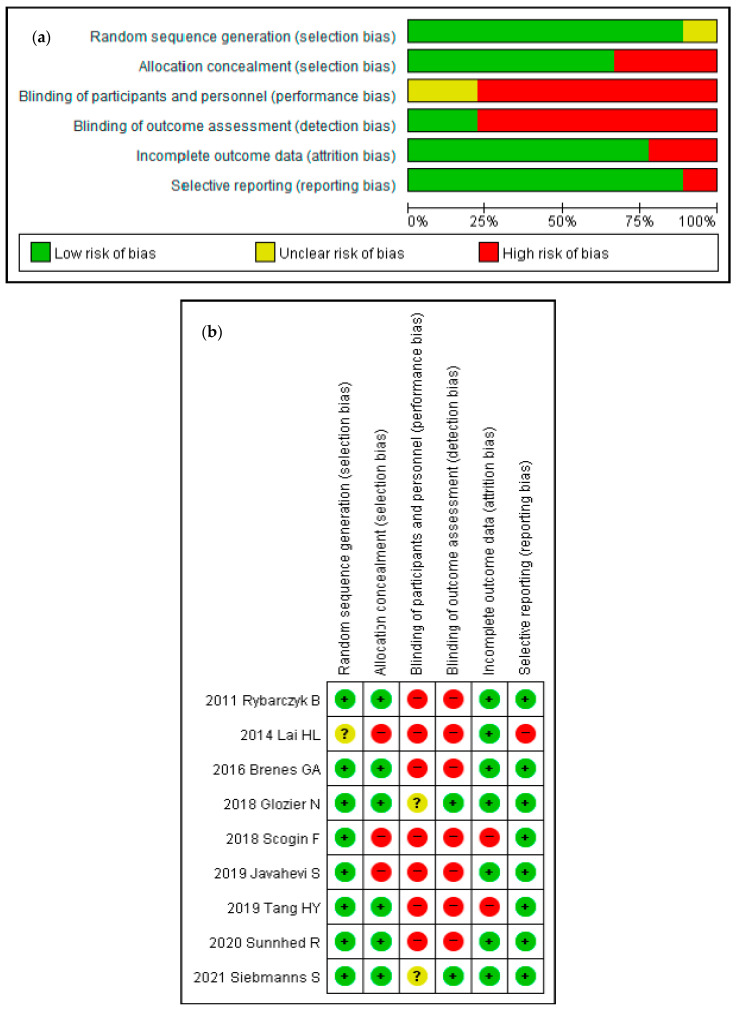
Quality assessment. (**a**) Risk of bias graph. (**b**) Risk of bias summary.

Of the sixteen selected studies, ten were RCTs, five were quasi-experimental studies, and one was a retrospective study. The ten RCTs assessed using the RoB tool showed a low risk of attrition and reporting bias. However, these studies showed a high risk of performance and detection bias. As for selection bias, the random sequence generation was low, but three of the ten studies showed high allocation concealment. Figure 2 presents the results of quality assessment using the RoB tool.

### 3.3. Study Outcomes

#### 3.3.1. Internet-Based CBT

This study considers most of the research on behavioral intervention using mobile health technology to improve the sleep of the elderly.

**Figure 3:**
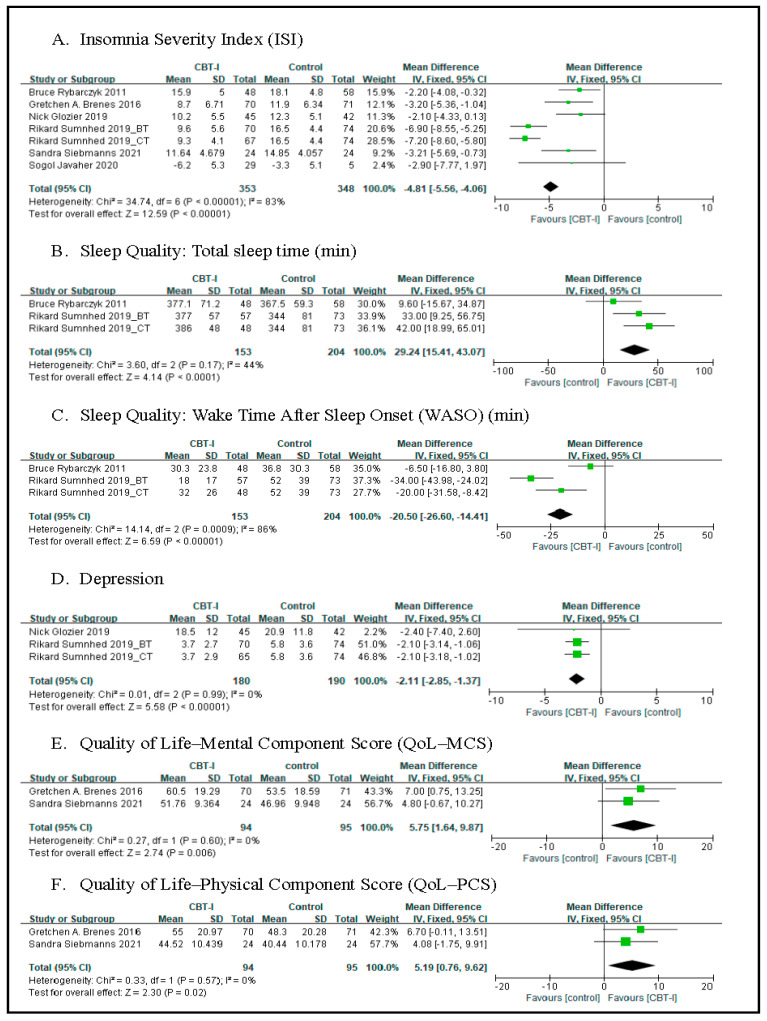
Meta-analysis of randomized control trial.

**Figure 4:**
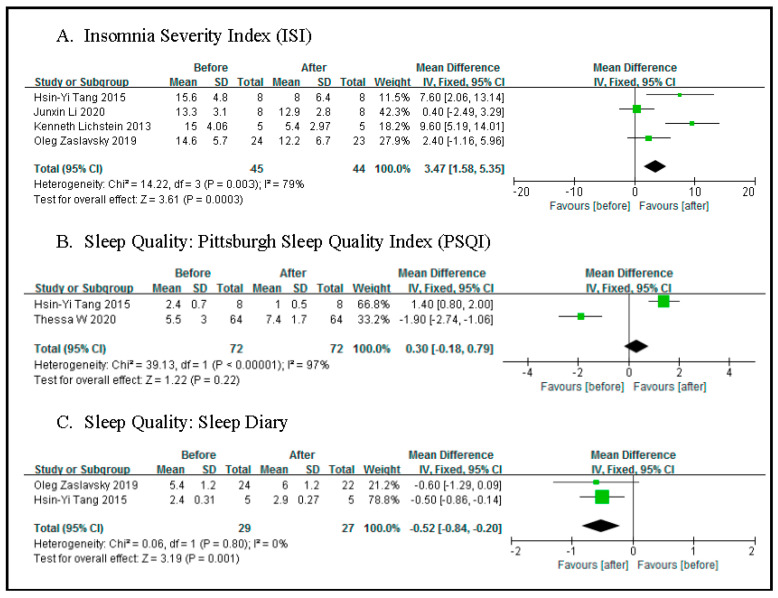
Meta-analysis of quasi-experimental studies.

ISI: Our meta-analysis of six RCTs showed a significant reduction in ISI in the CBT-I group compared to the control group (−4.81 (−5.56, −4.06), *p* < 0.00001, I^2^ = 83%) (Figure 3A). As for the four quasi-experimental studies, we find a significant reduction in ISI after the study (3.47 (1.58, 5.35), *p* = 0.0003) (see Figure 3A or Figure 4A).

Sleep Quality: Total sleep time (min)

Three RCTs showed significant improvement in total sleep time in the CBT-I group compared to the control group (29.24 (15.41, 43.07), *p* < 0.0001) (see Figure 3B).

Sleep Quality: Wake time after sleep onset (WASO) (min)

Meta-analysis results showed significantly reduced WASO for the CBT-I group compared to the control group (−20.50 (−26.60, −14.41), *p* <0.00001) (see Figure 3C).

Depression

This study examined the effects of integrated CBT on depression and insomnia, delivered through video conferences in middle-aged and elderly people with depression and insomnia, to obtain the following results. Cognitive-behavioral therapy for depression and insomnia (CBT-D + CBT-I) participants showed significant improvement in post-treatment sleep and three-month follow-up than usual care participants (study no. 635), while RCT meta-analysis results showed a significant reduction in depression in the CBT-I group compared to the control group (−2.11 (−2.85, −1.37), *p* < 0.00001, I^2^ = 0%) (see Figure 3D).

Quality of Life–Mental Component Score (QoL–MCS)

Meta-analysis results showed significant improvement in QoL–MCS for the CBT-I group compared to the control group (5.75 (1.64, 9.87), *p* = 0.006, I^2^ = 0%) (see Figure 3E).

Quality of Life–Physical Component Scores (QoL–PCS)

Meta-analysis results showed significant improvement in QoL–PCS for the CBT-I group compared to the control group (5.19 (0.76, 9.62), *p* = 0.02, I^2^ = 0%) (see Figure 3F).

Physical Activity

One study applied personalized physical activity training, real-time physical activity self-monitoring, interactive prompts and feedback with smartwatches, and telephone counseling with exercise trainers and research team members for four weeks to confirm positive changes in the level of physical activity of the elderly after intervention [15].

Others

One study considered the reduction in risk of coronary artery disease through insomnia treatment using web-based CBT-I. Another identified two self-help CBT-I effects in the elderly with osteoarthritis or coronary artery disease. Participants were randomly assigned to the book version or improved multimedia version of CBT-I; multimedia participants showed better improvement in three sleep measurements compared to book participants [16]. The details of these studies are presented in Tables S1 and S2.

#### 3.3.2. Audiovisual Stimulation (AVS)

Of the two studies using AVS on sleep, the first is as follows. After exposure to AVS for 30 min, the active group significantly increased their delta power compared to the control group, confirming controlled evidence that active AVS induction increased the delta quantitative electroencephalogram (QEEG) activity in people with insomnia and that these changes were immediate [17]. In another study performing neurofeedback AVS on eight elderly people, the severity of insomnia decreased to the sub-critical insomnia range after one month of AVS intervention. Significant changes were observed in the PSQI subscales of daytime dysfunction (*p* = 0.048) and sleep quality (*p* = 0.004), and the Patient Health Questionnaire-9 (PHQ-9) score decreased in five out of eight subjects with a reference score of 5 or more (*p* = 0.004) [18].

#### 3.3.3. Music Video Intervention

One study examined the intervention of soothing music for sleep improvement. This study was conducted in a sleep laboratory in a hospital with 38 subjects aged 50 to 75. The intervention was for 30 min to find significantly shorter sleep-mechanical delay time in the music video conditioned experimental group than in the normal-care condition group (*p* = 0.002) [19].

#### 3.3.4. Gight (Innovative Automated Guiding Light)

A study on applying guiding nightlight, an intervention method for the elderly’s fear of falling (FOF) and improving sleep quality, presents the following results. The study on night FOF and sleep quality by installing automatic LED strips in 64 participants’ homes showed that FOF fell from 5.5 ± 3.0 to 3.8 ± 3.2 (*p* = 0.001) and sleep quality increased from 6.7 ± 2.4 to 7.4 ± 1.7 (*p* = 0.012) [20].

## 4. Discussion

Sleep health is a public health priority, according to the Center for Disease Control’s Healthy People 2020 Report [21]. However, sleep disturbance has become a very common disease and is increasing, especially in the elderly [22]. Therefore, several ICT-based interventions have been made recently for elderly experiencing sleep disorders. Such interventions for sleep improvement can be made in various ways, thus making it necessary to evaluate the effectiveness of these methods in an integrated manner. In this study, we conduct a systematic review and meta-analysis of related studies in order to evaluate the effectiveness of ICT in addressing sleep issues of the elderly. We review the results of 16 analytical studies as outcome categories of ISI, sleep quality, physical activity, depression, and quality of life based on the indicators reported in each study.

**ICT-based intervention for ISI and sleep quality**

From our meta-analysis, we find that ICT-based interventions are effective in managing sleep disturbances of older adults. Specifically, ISI was lower and sleep quality (total sleep time) was higher in the experimental group than the control group, as confirmed in this study and in a few systematic reviews of studies conducted on sleep disturbances of the elderly through ICT intervention. From a systematic review of ICT interventions for elderly persons with insomnia reported in 2019, we summarize 11 findings of interventional methods as follows: (1) Internet-delivered CBT-I, (2) virtual coach, and (3) sleeping technology [23]. This systematic review shows that Internet-based CBT-I programs are useful and an easy first step in treating insomnia in the elderly. The results are the same as those of this study. The American Academy of Sleep Medicine has strongly recommended CBT for insomnia and medication when CBT is ineffective or impossible. In other words, CBT is strongly recommended as the first treatment for insomnia [24]. Supporting these guidelines, this study confirms that ICT-based CBT is effective for older people experiencing sleep disorders. Therefore, we propose the various ICT methods used in the reviewed studies for intervention in sleep disorders of the elderly.

**ICT-based intervention for physical activity**

As for physical activity, Muellmann et al. [12] reviewed some studies using eHealth tools for the physical activity of older adults. This review evaluated the effectiveness of eHealth interventions (i.e., those accessible via computers, portable devices, phones, smartphones, or tablets) and included RCTs or quasi-experimental studies comparing eHealth and non-eHealth interventions in physical activities of adults aged 55 and older. From the review results, 20 studies (over 25 publications) found eHealth interventions effective in promoting physical activity among adults aged 55 years and older. Another systematic review of experimental studies (clinical trials and RCTs) published between 2012 and 2016 to assess the effectiveness of mobile health (mHealth) in changing health behavior and improving the disease recovery of older adults shows similar results [13]. The review focused widely on health behaviors ranging from physical activity, eating habits, and sleep to medication compliance and mental health outcomes. The authors of 12 studies found evidence supporting the effectiveness of various mHealth tools (cell phones, text messaging, mobile apps) in improving various health and behavioral outcomes.

**ICT-based intervention for depression and quality of life**

ICT-based intervention methods are found effective in most cases, as confirmed in this study, with positive effects on depression, quality of life, and other indicators. A study on CBT provided over the Internet for depression of the elderly found a significant short-term effect [25]. In addition, a study evaluating the quality of life considered the general health perceptions, pain, physical functioning, and role limitations due to physical health problems [26]. If an intervention to improve sleep can positively affect the quality of life, this can be the basis for interventions to improve sleep, as sleep improves the quality of life. Of course, we need further assessments of the effectiveness of ICT-based intervention in improving depression and quality of life. Another systematic review [27] found ICT-based CBT effective in improving the sleep of adults with insomnia, suggesting that efforts should be made to educate the public and expand access to this mode of treatment.

**Various ICT-based interventions for sleep disorder**

Next, we consider the specific interventional methods used in each study. In recent years, we find considerable innovation in ICT applications to support the health care of elderly persons [23]. As the name suggests, ICT uses three terms: information, communication, and technology. Information refers to the data users’ access, while communication denotes the connection between two parties. Technology refers to the technical components used in ICT to access and communicate information, such as computers, notebooks, internet, LAN, and video conferencing cameras [28]. Most of the interventions are web-based and use the Internet, while some methods have combined advanced technologies, such as mobile applications, smartwatches, and wearable devices. One interventional study used AVS devices (MindPlace Procyon) with LED goggles, while others used the telephone and Skype or an innovative intervention method combining technologies utilizing multimedia and music videos to develop the automated guiding light named “Gight.” Among the factors confirmed to be effective in various studies, some may have been developed for the elderly to be applied in an easy-to-use and simple manner. Therefore, this point should be considered when considering ICT intervention for the elderly.

**ICT-based intervention and healthcare professional’s role**

ICT has become so inevitable, with people dependent on technology, that no one can imagine living without it [28,29,30]. In this regard, technology has revolutionized the way nurses meet the needs of older persons, families, and communities [15,31,32]. A few studies related to ICT intervention methods for healthcare professionals, including nurses, motivated participants to complete the I-CBT program with appreciative, confirmatory, advisory, and reflective support from nurses, to obtain improved sleep-related indicators [15]. Thus, positive results could be obtained through the pivotal role of healthcare professionals in ICT intervention. Human reliance on ICT-based technology can be expected to increase in many areas in the future. It will be the same in the field of health maintenance and improvement, and innovative methods using ICT grafting will be developed and applied differently every day. As medical services move from acute treatment environments to community-based environments, nursing education and practices will have to change so that nurses might play a pivotal role in reliable evidence-based practices to promote healthy behaviors such as good sleep and physical activity of the elderly.

### Strengths and Limitations

The main strength of this review is that it is the most up-to-date and comprehensive quantitative synthesis of existing data on various ICT-based interventions, including Internet-based CBT-I for the elderly. In particular, we explored the effectiveness of Internet-based CBT among older adults to find that it has a positive effect on indirect factors such as depression, quality of life, physical activity, and sleep-related outcomes. Therefore, a major contribution of this work is identifying the steps to be taken to enhance the effectiveness of sleep interventions.

Regarding the limitations of this study, this review revealed a wide variation in the methodology of sleep disturbance programs used across studies. Thus, we found heterogeneity in some meta-analyses, wide-ranging confidence intervals, and unbalanced sample sizes in the experimental and control groups. However, although heterogeneity was high, the CBT-I intervention seemed to be effective, as all studies showed the same direction, which was that the result was consistently effective. Second, because the follow-up period of most studies was not long, the present results should be interpreted with caution, and more RCTs with large sample sizes and comparative studies utilizing long-term follow-up data are needed to evaluate the effectiveness of ICT intervention for insomnia. Another limitation is that only studies published in English or Korean were used for our analysis. Finally, due to the limited number of studies pooled in each meta-analysis, funnel plots were not generated, and therefore publication bias cannot be discounted.

## 5. Conclusions

Sleep disturbance is a very common disease that is increasing, especially among the elderly. ICT-based interventions for sleep improvement can be made in several ways. This study has confirmed that such interventions effectively improve the quality of sleep and reduce sleep disturbances in the elderly. ICT interventions such as Internet-based CBT, AVS, or automated guiding light can be applied for the treatment of insomnia among the elderly. Internet-based CBT has a positive effect on indirect factors such as depression, quality of life, and physical activity, as well as sleep-related outcomes. Nursing education and practices in community-based environments should be adjusted to allow nurses to play a pivotal role in evidence-based ICT interventions in promoting better health behaviors such as good sleep of the elderly.

## Data Availability

Publicly available datasets were analyzed in this study. This data can be found here: [DOI 10.17605/OSF.IO/KMRUA].

## Supplementary Materials

The following are available online at https://www.mdpi.com/article/10.3390/s21186003/s1, Table S1: General characteristics of selected articles, Table S2: PRISMA 2009 Checklist.

## Abbreviations

CBTi	Cognitive behavioral therapy for insomnia
CCBT-I	Computerized CBTi
ICT	Information and communication technologies
mHealth	Mobile health
PSQI	Pittsburgh sleep quality index
RCTs	Randomized controlled trials
SHUTi	Sleep healthy using the Internet
KHIDI	Korea Health Industry Development Institute

## References

[1] Streatfeild J., Smith J., Mansfield D., Pezzullo L., Hillman D. (2021). The Social and Economic Cost of Sleep Disorders. Sleep.

[2] Chattu V.K., Manzar M.D., Kumary S., Burman D., Spence D.W., Pandi-Perumal S.R. (2018). The Global Problem of Insufficient Sleep and Its Serious Public Health Implications. Healthcare.

[3] Saconi B., Polomano R.C., Compton P.C., McPhillips M.V., Kuna S.T., Sawyer A.M. (2021). The influence of sleep disturbances and sleep disorders on pain outcomes among veterans: A systematic scoping review. Sleep Med. Rev..

[4] Ruiz-Castell M., Makovski T.T., Bocquet V., Stranges S. (2019). Sleep duration and multimorbidity in Luxembourg: Results from the European Health Examination Survey in Luxembourg, 2013–2015. BMJ Open.

[5] Stranges S., Tigbe W., Gómez-Olivé F.X., Thorogood M., Kandala N.-B. (2012). Sleep problems: An emerging global epidemic? Findings from the INDEPTH WHO-SAGE study among more than 40,000 older adults from 8 countries across Africa and Asia. Sleep.

[6] Cheng S.K., Dizon J. (2012). Computerised Cognitive Behavioural Therapy for Insomnia: A Systematic Review and Meta-Analysis. Psychother. Psychosom..

[7] Zachariae R., Lyby M.S., Ritterband L.M., O’Toole M.S. (2016). Efficacy of internet-delivered cognitive-behavioral therapy for insomnia—A systematic review and meta-analysis of randomized controlled trials. Sleep Med. Rev..

[8] Pew Research Center (2014). Older Adults and Technology Use. http://www.pewinternet.org/2014/04/03/older-adults-and-technology-use/.

[9] Pew Research Center (2017). 17 Striking Findings from 2017. https://www.pewresearch.org/fact-tank/2017/12/26/17-striking-findings-from-2017/.

[10] Eurostat (2016). Internet Access and Use Statistics—Households and Individuals—Statistics Explained. http://ec.europa.eu/eurostat/statisticsexplained/index.php/Internet_access_and_use_statistics_-households_and_individuals#More_than_four_fifths_of_Europeans_used_the_internet_in_2016.

[11] Pew Research Center (2016). 16 Striking Findings from 2016. https://www.pewresearch.org/fact-tank/2016/12/21/16-striking-findings-from-2016/.

[12] Muellmann S., Forberger S., Möllers T., Zeeb H., Pischke C.R. (2016). Effectiveness of eHealth interventions for the promotion of physical activity in older adults: A systematic review protocol. Syst. Rev..

[13] Changizi M., Kaveh M.H. (2017). Effectiveness of the mHealth technology in improvement of healthy behaviors in an elderly population-a systematic review. Mhealth.

[14] Collaboration T.C. (2020). Review Manager (RevMan). Version 5.4 [Computer Program]. https://training.cochrane.org/online-learning/core-software-cochrane-reviews/revman.

[15] Li J., Hodgson N., Lyons M.M., Chen K.C., Yu F., Gooneratne N.S. (2020). A personalized behavioral intervention implementing mHealth technologies for older adults: A pilot feasibility study. Geriatr. Nurs..

[16] Rybarczyk B., Mack L., Harris J.H., Stepanski E. (2011). Testing two types of self-help CBT-I for insomnia in older adults with arthritis or coronary artery disease. Rehabil. Psychol..

[17] Tang H.J., McCurry S.M., Riegel B., Pike K.C., Vitiello M.V. (2019). Open-Loop Audiovisual Stimulation Induces Delta EEG Activity in Older Adults With Osteoarthritis Pain and Insomnia. Biol. Res. Nurs..

[18] Tang H.Y., Vitiello M.V., Perlis M., Riegel B. (2015). Open-Loop Neurofeedback Audiovisual Stimulation: A Pilot Study of Its Potential for Sleep Induction in Older Adults. Appl. Psychophysiol. Biofeedback.

[19] Lai H.L., Chang E.T., Li Y.M., Huang C.Y., Lee L.H., Wang H.M. (2015). Effects of music videos on sleep quality in middle-aged and older adults with chronic insomnia: A randomized controlled trial. Biol. Res. Nurs..

[20] Tholking T.W., Lamers E.C.T., Olde Rikkert M.G.M. (2020). A Guiding Nightlight Decreases Fear of Falling and Increases Sleep Quality of Community-Dwelling Older People: A Quantitative and Qualitative Evaluation. Gerontology.

[21] Redline S., Purcell S.M. (2021). Sleep and Big Data: Harnessing data, technology, and analytics for monitoring sleep and improving diagnostics, prediction, and interventions—An era for Sleep-Omics?. Sleep.

[22] Lee E. (2020). Cognitive behavior therapy for insomnia. J. Korean Med. Assoc..

[23] Salvemini A., D’Onofrio G., Ciccone F., Greco A., Tullio A., Addante F., Sancarlo D., Vendemiale G., Serviddio G., Ricciardi F. (2019). Insomnia and Information and Communication Technologies (ICT) in Elderly People: A Systematic Review. Med. Sci..

[24] Kim S.J. (2020). Recent Advances in Diagnosis and Treatment of Insomnia Disorder. JKANA.

[25] Glozier N., Christensen H., Griffiths K.M., Hickie I.B., Naismith S.L., Biddle D., Overland S., Thorndike F., Ritterband L. (2019). Adjunctive Internet-delivered cognitive behavioural therapy for insomnia in men with depression: A randomised controlled trial. Aust. N. Z. J. Psychiatry.

[26] Brenes G.A., Danhauer S.C., Lyles M.F., Anderson A., Miller M.E. (2016). Effects of Telephone-Delivered Cognitive-Behavioral Therapy and Nondirective Supportive Therapy on Sleep, Health-Related Quality of Life, and Disability. Am. J. Geriatr. Psychiatry.

[27] Seyffert M., Lagisetty P., Landgraf J., Chopra V., Pfeiffer P.N., Conte M.L., Rogers M.A. (2016). Internet-Delivered Cognitive Behavioral Therapy to Treat Insomnia: A Systematic Review and Meta-Analysis. PLoS ONE.

[28] Sharma D.M. (2021). Influence of ICT and Its Dynamic Change in Daily Life of Human Being. J. Contemp. Issues Bus. Gov..

[29] Pew Research Center October 2019, Experts Optimistic about the Next 50 Years of Digital Life. https://www.pewresearch.org/internet/wp-content/uploads/sites/9/2019/10/PI_2019.10.28_The-Next-50-Years-of-Digital-Life_FINAL.pdf.

[30] Fahn M., Yan S. (2021). Analysis of the Impact of 5G Development on the Macroeconomy. Adv. Soc. Sci. Educ. Humanit. Res..

[31] Jahnke I., Riedel N., Popescu M., Skubic M., Rantz M. (2021). Social practices of nurse care coordination using sensor technologies-Challenges with an alert system adoption in assisted living communities for older adults. Int. J. Nurs. Sci..

[32] Rutledge C.M., Gustin T. (2021). Preparing Nurses for Roles in Telehealth: Now is the Time!. Online J. Issues Nurs..

