# Supplementary material for: Effectiveness of Information and Communication Technology (ICT) Interventions in Elderly’s Sleep Disturbances: A Systematic Review and Meta-Analysis
Source: Sensors (Basel). 2021 Sep 8;21(18):6003. doi: 10.3390/s21186003 (PMC8468949; doi:10.3390/s21186003)
Supplement: Supplementary file 1 [file sensors-21-06003-s001.zip › Table S1(Supplementary)2.pdf]

Table S1. General characteristics of selected articles.

Author(s) (Year)	Country	Study Design	Participant Characteristics N(I/C)	Age (mean ± SD)	Women n (%)	Selection Criteria	Times / Week, Session Duration, and Period	Follow Up / Measurement	Intervention Method: Intervention Group	Control Group
Siebmanns, Johansson	Sweden	RCT	48(24/24)	T:72.53±9.81<br>I:72.52±9.81<br>C:72.58±10.37		Inclusion criteria<br>• Over 18 years<br>• Verified diagnosis of insomnia<br>• Verified CVD (cardiovascular disease) diagnosis<br>Exclusion criteria<br>• No access to the Internet, computer, or smartphone<br>• The need to understand Swedish, etc.	• 9 weeks	• 6-month<br>• The ISI<br>• The Short Form Health Survey 12<br>• Visual inspection<br>• Emailed a link to complete a web-based questionnaire (on demographics, sleep quality, and physical and mental health)	• I-CBTI (internet cognitive behavioral therapy) program<br>• Nine modules<br>1. Introduction<br>2. living with cardiac disease<br>3. sleep<br>4. heart disease<br>5. sleep problems<br>6. stimulus control<br>7. sleep restriction<br>8. thoughts that contribute to sleeping badly<br>9. stress related to heart disease that can contribute to sleep problems, completion.<br>• 20 assignments in the 9 modules I-CBTI intervention<br>• Feedback	• Self-study program<br>• Three modules<br>1. introduction<br>2. living with cardiac disease<br>3. sleep<br>• No assignments<br>• No support by nurses
Javaheri, Reid	USA	RCT	29(15/14)	I:70.3±10.0<br>C:72.9±9.2		Inclusion criteria<br>• ISI score over 10<br>• Patient Health Questionnaire-8 under 15<br>• At least 3 months of insomnia symptoms<br>• Aged over 18	• 6 weeks	• The ISI-5<br>• The Patient Health Questionnaire-8<br>• Epworth Sleepiness Score<br>• Duke Health Profile	wCBT-i and general sleep education<br>• wCBT-I (web-based cognitive behavioral therapy for insomnia)<br>• instructions on the use of the Internet program<br>• Go! To sleep (educational multicomponent cognitive behavioral therapy program)<br>• Stimulus control<br>• Sleep restriction therapy<br>• Sleep hygiene<br>• Cognitive therapy and	general sleep education alone<br>• counseled on how to access online sleep diaries

										relaxation therapy<br>• Email reminding	
Glozier, Christensen	Australian and New Zealand	RCT	87(45/47)	I:58.6±6.3<br>C:58.1±6.1		• Aged over 50<br>• Meet diagnostic criteria for a current depressive disorder, major depressive disorder (MDD), or dysthymia, with insomnia symptoms	• min<br>• weeks	30-40<br>12	• 12 week and 6 months<br>• The self-report Centre for Epidemiological Studies Depression Scale (CES-D)<br>• The ISI<br>• State-Trait Personality Inventory<br>• Online survey	Active treatment arm (SHUTi)<br>• Incorporating sleep restriction<br>• Stimulus control<br>• Sleep hygiene<br>• Cognitive restructuring<br>• Relapse prevention<br>• Clinical assessment	Attention-control arm (online psychoeducation)<br>• Non-tailored<br>• Standard sleep health promotion material<br>• Environmental and behavioral strategies to improve sleep<br>• Clinical assessment
McCurry, Von Korff	USA	RCT	327(163/164)			• Identify across Washington State<br>• Diagnosis of osteoarthritis on at least one health care visit<br>• Aged over 60<br>• Continuously enrolled at KPW for at least 1 year	• times over weeks<br>• months	6<br>8<br>2	• 12-month<br>• The ISI<br>• The Brief Pain Inventory-short form<br>• The Patient Health Questionnaire<br>• The Flinders Fatigue Scale<br>• Estimate the proportion of individual's experience of reduction in insomnia severity and relative proportion	6-session telephone-delivered CBT-I intervention<br>• 1. Information on insomnia and sleep behavioral/scheduling: Stimulus control<br>• 2. Behavioral/scheduling: Bed restriction<br>• 3. Information on sleep and aging<br>• 4. Constructive worry Relaxation/mindfulness<br>• 5. Sleep hygiene recommendations Adopting beliefs/attitudes that foster good sleep<br>• 6. Review of behavioral and cognitive strategies Maintaining treatment gains/relapse prevention<br>• Contact with a CBT-I interventionist	6-session telephone education only control condition (EOC)<br>• Well-accepted group EOC condition<br>• 1. Introduce to OA pain and sleep: Sleep hygiene strategies<br>• 2. Tools for protecting your joints: stages of sleep<br>• 3. Prescription medication treatments for pain and sleep<br>• 4. Exercise for pain and sleep<br>• 5. Using nutrition to help reduce pain and improve sleep<br>• 6. Over-the-counter medication and supplements<br>• Contact with a CBT-I interventionist
Scogin, Lichstein	USA	RCT	40(22/18)	I:58.32±6.69<br>C:59.78±8.5	I:18(81.8)<br>C:18(100)	• Aged over 50<br>• Resident of Alabama's Black Belt	• min<br>• sessions	25-<br>10	• 3-month<br>• Sociodemographic questionnaire<br>• The Saint Louis University Mental	10-session of CBT-D + CBT-I<br>• CBT-D: cognitive-behavioral therapy for depression<br>• CBT-I: cognitive-behavioral therapy for insomnia	Usual care<br>• Physician-recommended primary care services

					• Absence of significant cognitive impairment<br>• Absence of suicidality, self-reported, other sleep disorders such as apnea<br>• Concurrence from patient's primary care		Status Examination (The SLUMS)<br>• The Hamilton Depression Rating (The HAM-D)<br>• The ISI<br>• A telephone interview		
Sunnhed and Jansson Frojmark	Sweden	RCT	219 - *3 groups* (72/73/74)	I:51.5±12.5<br>I:51.8±14.5<br>C:54.2±14.6	I:55(76.4)<br>I: 51(69.9)<br>C:54(73)	• Register themselves on the Internet platform<br>• Web-based screening questionnaire<br>• Occurrence of sleeping difficulty three nights or more per week in at least 3 months	• 15 min per week<br>• 10 weeks	• 6-month<br>• The Mini-International Neuropsychiatric interview<br>• Diagnostic measures at pretreatment<br>• Treatment credibility during the first week of therapy<br>• Primary outcome biweekly from pre- to post-treatment	CT: cognitive therapy and consisted of sleep-interfering or sleep-related worry, unhelpful beliefs about sleep, attentional bias and monitoring for sleep-related threat, misperception of sleep, and safety behaviors<br>BT: behavioral therapy and consisted of sinus rhythm and sleep hygiene<br>Treatment<br>• Telephone support<br>• Feedback<br>• Help to problem solve the issue<br>• Ended with the delivery of the next internet module	WL: waitlist
Brenes, Danhauer	USA	RCT	141(70/71)		T:115(81.6)<br>I:58(83.9)<br>C:57(80.3)	• Aged over 60<br>• Principal or co-principal diagnosis of generalized anxiety disorder<br>• Resident of rural countries within North Carolina	• 10 weekly sessions<br>• 2,4,8, and 12 weeks (booster session)	• 12-month<br>• The ISI<br>• The SK-36(self-report)<br>• The Pepper Center Tool for Disability	CBT-I<br>• 11 weekly psychotherapy session<br>• recognition of anxiety symptoms<br>• relaxation<br>• cognitive restructuring<br>• the use of coping statements,<br>• problem-solving<br>• worry control<br>• behavioral activation<br>• exposure therapy	NST-T<br>• 10 weekly sessions<br>• high-quality therapeutic relationships warm genuine and accepting atmosphere through the use of supportive and reflective communications

								• relapse prevention.	
Rybarczyk, Mack	USA	RCT	106(58/48)	T:68<br>I:67.9±7.5<br>C:67.2±8.5	I:39<br>C:33	• Aged over 55<br>• At least three episodes of insomnia per week for at least 6 months<br>• Daytime consequences of insomnia, such as fatigue, irritability, or difficulty concentrating<br>• Self-report of a physician-verified diagnosis for the OA or CAD	• one module per week<br>• 8-week<br>• 15-min (telephone support weekly)	• 1 year follow up<br>•<br>• Paper-and-pencil records<br>• Pittsburgh Sleep Quality Index<br>• Sleep Impairment Index<br>• Dysfunctional Beliefs and Attitudes about Sleep Scale	Enhanced multimedia version of CBT-I<br>• Mailed<br>• Treatment kit (self-help guide book)<br>• Two videotapes<br>• Audiotape<br>• Weekly telephone support	Book version<br>• Mailed<br>• Enlarged copied version of the 207-page out-of-print book<br>• Audiotape<br>• Weekly telephone support
Chung, Kim	Republic Of Korea	retrospective	40(40/9)	I:75.75±5.87<br>C:71.56±4.36	I: 35(88)<br>C: 9(100)	• Senior residents in Seodaemun-gu<br>• Aged over 60 years<br>• Interest in smartphone-based self-help treatment for insomnia<br>• Adequate literacy	• 2-hour 1-day (training program)<br>• 1 week (self-help)	• 8-week<br><br>• The Korean version of the Geriatric Depression Scale-15<br>• The Subjective Memory Complaints Questionnaire<br>• The Pittsburgh Sleep Quality Index<br>• Perceived difficulty questionnaire<br>• Usefulness, Satisfaction, and Ease of Use Questionnaire<br><br>• Interview<br>• Complete the pencil-and-paper questionnaires on sociodemographic characteristics and subjective evaluation of	1-day training program for MIND MORE use<br>• Hands-on experience with the MIND MORE app with the help of therapist and volunteer workers<br>MIND MORE App<br>• Sleep hygiene education program<br>• Quiz session<br>• Sleep diary<br>• Thought record and constructive worry worksheet<br>• Learning progress management<br>• Clipping button<br>List of clipped pages	1-week self-help intervention with MIND MORE

								mental health status and usability of the MIND MORE	
Li, Hodgson	USA	quasi-experimental (before-after)	8 - *1 groups*	T:74±5.42		• Age (65-85)<br>• No prior diagnosis of dementia<br>• Sedentary lifestyle<br>• Poor sleep quality<br>• No diagnosis of sleep apnea	• y<br>• week	weekl<br>4-	• The Physical Activity Scale for the Elderly (PASE)<br>• The Insomnia Sleep Index (ISI)<br>• Epworth Sleeping Scale (ESS)<br>• The Montreal Cognitive Assessment (MoCA)<br>• Actiwatch 2(small watch-like device worn on the wrist)<br>• Sleep diary	• Technology learning session<br>• A 2-hour personalized physical activity educational session<br>• Physical activity self-monitoring<br>• Phone consultation<br>• Financial incentives
Zaslavsky, Thompson	USA	quasi-experimental (before-after)	24	T:71.0±4.0	T:17(70)	• Aged over 65<br>• Having a diagnosis of OA<br>• Having a telephone<br>• Having physical guidelines level below the Rapid Assessment of Physical Activity scale	•<br>week	14-	• 5-week<br>• The Graded chronic Pain Scale<br>• Self-efficacy<br>• The ISI<br>• Acceptance of Sleep Difficulties<br>• The iCardia research platform<br>• Daily step count<br>• The Fitbit Charge 2(wristband)	• 14 weeks study staff routine contact<br>• Personalized reminders to sync their activity device with the app<br>• Wearable activity tracking devices<br>• Personalized texts<br>• Motivational interviews<br>• Remote and self-monitoring
Lichstein, Scogin	USA	quasi-experimental (before-after)	5	T:65.8±10.4	T:4(80)	• Aged over 50<br>• Resident of the black belt or adjacent countries<br>• Absence of significant cognitive impairment and other sleep disorders	• mins weekly session<br>• sessions	10-50<br>10	• 2-month<br>• Sociodemographic information<br>• Saint Louis University Mental Status Examination (SLUMS)<br>• Consensus Sleep Diary<br>• ISI<br>• Hamilton	Individual therapy using real-time audio and visual computer-based communication<br>• 1-5 sessions- insomnia and depression treatment<br>• 5-10 sessions- depression treatment<br>• Insomnia treatment included sleep hygiene, sleep compression, stimulus control, and

								Rating Scale for Depression<br>• Working Alliance Inventory-observer Rating Form<br>• Skype (a voice over internet protocol application)	passive relaxation<br>• Depression treatment was the cognitive-behavioral mediational model, behavioral activation, and identification ad disputation of unhelpful thoughts	
Tang, McCurry	USA	RCT	16(9/7)	I:67.2±5.0<br>C:69.6±4.4	I:9 (100)<br>C:6 (85.7)	Inclusion criteria<br>• Aged over 60<br>• Have difficulty sleeping over the past 3 months<br>• Have OA pain	• 5 min eye closed and 30 min AVS<br>• 2 weeks	• The ISI<br>• PSQI<br>• Brief pain inventory-short form<br>• Patient health questionnaire<br><br>• AVS device (a pocket-sized player with standard headphone and LED goggles)	Active<br>• Test the sleep-promotion effects of a 30-min AVS (open-loop audiovisual stimulation) program<br>• 30-min pulsing light (red and green spectrum) and pulsing binaural sounds from 10 Hz to 2 Hz<br>• The protocol consisted of stimulation from 10 to 6 Hz during the first 3 min, then 6-3 Hz at 4-28 min, and 3-2 Hz at 29-30 min	Placebo-control<br>• Placebo-control AVS program consisted of 30 min of constant dim light that slowly changed in color and a steady monotone at an ultralow frequency<br>The protocol consisted of stimulation from 1 to 0.4 Hz during the first 3 min, the 0.4-0.2 Hz at 4-6 min, and 0.2 Hz at 7-30 min
Tholking, Lamers	Netherlands	quasi-experimental (before-after)	64	T:81±8.0	T:42(66)	• Aged over 65<br>• Toileting independently at night<br>• Ability to generate the outcomes	• 118±5<br>7 days	• Single-item overnight forced oscillation Technique (FOF)<br>• Single-item sleep quality, self-reported fall rate<br>• Functional Electrical Stimulation<br>• Pittsburgh Sleep Quality Index	Gight (innovative automated guiding light)<br>• Consisted of an in length adjustable LED strip(12V) attached to the baseboard, activated by a motion sensor, causing it to light up for 30 s after motion<br>• 1 long LED strip was installed in a room on the path generally walked from bed to bathroom	
Lai, Chang	Taiwan	RCT	38	T:59.61±6.71	T: 33(86.8)	• Experienced insomnia<br>• Aged 50-75<br>• Taiwanese<br>• Usual-care routine before bedtime	• 30- min<br>• 3 nights	• Pittsburgh Sleep Quality Index<br>• Visual Analog Scale<br>• Polysomnography<br>• State-Trait Anxiety Inventory<br>• Short-form	Viewing/listening to an MV intervention > usual care<br>• Watching a peaceful Buddhist MV before bedtime<br>• During MV invention night participants were asked to turn off their mobile phones<br>• Participants watched the MV on a television in their room	Usual care > MV intervention<br>• Two-way communications<br>• Temperature ranged from 24-26<br>• Background noise levels were 30-35 dB

								Geriatric Depression Scale	while seated in a chair<br>• After watching the MV, participants went straight to bed
Tang, Vitiello	USA	quasi-experimental (before-after)	T:88±8.7	T:(88)	• Aged over 65<br>• Evidence of insomnia with a score of 8 or higher<br>• English speaking<br>• Sufficiently intact cognitively to participate	• min	30	• Multivariable Apnea Prediction Index<br>• International Restless Legs Syndrome Scale<br>• The ISI<br>• Sleep diary<br>• Pittsburgh Sleep Quality Index<br>• Patient Health Questionnaire-9	• The 30-min open-loop AVS intervention was delivered through a Walkman-sized device<br>• Test 30-min AVS program from 8 Hz to 1 Hz

< Included studies >

1. Brenes, G.A.; Danhauer, S.C.; Lyles, M.F.; Anderson, A.; Miller, M.E. Effects of Telephone-Delivered Cognitive-Behavioral Therapy and Nondirective Supportive Therapy on Sleep, Health-Related Quality of Life, and Disability. *Am. J. Geriatr. Psychiatry* **2016**, *24*, 846–854, http://doi.org/10.1016/j.jagp.2016.04.002.
2. Chung, K.; Kim, S.; Lee, E.; Park, J.Y. Mobile App Use for Insomnia Self-Management in Urban Community-Dwelling Older Korean Adults: Retrospective Intervention Study. *JMIR Mhealth Uhealth* **2020**, *8*, e17755, http://doi.org/10.2196/17755.
3. Glozier, N.; Christensen, H.; Griffiths, K.M.; Hickie, I.B.; Naismith, S.L.; Biddle, D.; Overland, S.; Thorndike, F.; Ritterband, L. Adjunctive Internet-delivered cognitive behavioural therapy for insomnia in men with depression: A randomised controlled trial. *Aust. N. Z. J. Psychiatry* **2019**, *53*, 350–360, http://doi.org/10.1177/0004867418797432.
4. Javaheri, S.; Reid, M.; Drerup, M.; Mehra, R.; Redline, S. Reducing Coronary Heart Disease Risk Through Treatment of Insomnia Using Web-Based Cognitive Behavioral Therapy for Insomnia: A Methodological Approach. *Behav. Sleep Med.* **2020**, *18*, 334–344, http://doi.org/10.1080/15402002.2019.1584896.
5. Lai, H.L.; Chang, E.T.; Li, Y.M.; Huang, C.Y.; Lee, L.H.; Wang, H.M. Effects of music videos on sleep quality in middle-aged and older adults with chronic insomnia: A randomized controlled trial. *Biol. Res. Nurs.* **2015**, *17*, 340–347, https://doi.org/10.1177/1099800414549237.
6. Li, J.; Hodgson, N.; Lyons, M.M.; Chen, K.C.; Yu, F.; Gooneratne, N.S. A personalized behavioral intervention implementing mHealth technologies for older adults: A pilot feasibility study. *Geriatr. Nurs.* **2020**, *41*, 313–379, http://doi.org/10.1016/j.gerinurse.2019.11.011.
7. Lichstein, K.L.; Scogin, F.; Thomas, S.J.; Dinapoli, E.A.; Dillon, H.R.; McFadden, A. Telehealth cognitive behavior therapy for co-occurring insomnia and depression symptoms in older adults. *J. Clin. Psychol.* **2013**, *69*, 1056–1065, http://doi.org/10.1002/jclp.22030.
8. McCurry, S.M.; Von Korff, M.; Morin, C.M.; Cunningham, A.; Pike, K.C.; Thakral, M.; Wellman, R.; Yeung, K.; Zhu, W.; Vitiello, M.V. Telephone interventions for co-morbid insomnia and osteoarthritis pain: The OsteoArthritis and Therapy for Sleep (OATS) randomized trial design. *Contemp. Clin. Trials* **2019**, *87*, 105851, doi:10.1016/j.cct.2019.105851.
9. Rybarczyk, B.; Mack, L.; Harris, J.H.; Stepanski, E. Testing two types of self-help CBT-I for insomnia in older adults with arthritis or coronary artery disease. *Rehabil. Psychol.* **2011**, *56*, 257–266, https://doi.org/10.1037/a0025577.

10. Scogin, F.; Lichstein, K.; DiNapoli, E.A.; Woosley, J.; Thomas, S.J.; LaRocca, M.A.; Byers, H.D.; Mieskowski, L.; Parker, C.P.; Yang, X.; et al. Effects of integrated telehealth-delivered cognitive-behavioral therapy for depression and insomnia in rural older adults. *J. Psychother. Integr.* **2018**, *28*, 292–309, http://doi.org/10.1037/int0000121.
11. Siebmanns, S.; Johansson, P.; Ulander, M.; Johansson, L.; Andersson, G.; Brostrom, A. The effect of nurse-led Internet-based cognitive behavioural therapy for insomnia on patients with cardiovascular disease: A randomized controlled trial with 6-month follow-up. *Nurs. Open* **2021**, *8*, 1755–1768, http://doi.org/10.1002/nop2.817.
12. Sunnhed, R.; Frojmark, M.J. Comparing internet-delivered cognitive therapy and behavior therapy for insomnia disorder: A randomized controlled trial. *Sleep Med.* **2017**, *40*, e319.
13. Tang, H.J.; McCurry, S.M.; Riegel, B.; Pike, K.C.; Vitiello, M.V. Open-Loop Audiovisual Stimulation Induces Delta EEG Activity in Older Adults With Osteoarthritis Pain and Insomnia. *Biol. Res. Nurs.* **2019**, *21*, 307–317, https://doi.org/10.1177/1099800419833781.
14. Tang, H.Y.; Vitiello, M.V.; Perlis, M.; Riegel, B. Open-Loop Neurofeedback Audiovisual Stimulation: A Pilot Study of Its Potential for Sleep Induction in Older Adults. *Appl. Psychophysiol. Biofeedback* **2015**, *40*, 183–188, https://doi.org/10.1007/s10484-015-9285-x.
15. Tholking, T.W.; Lamers, E.C.T.; Olde Rikkert, M.G.M. A Guiding Nightlight Decreases Fear of Falling and Increases Sleep Quality of Community-Dwelling Older People: A Quantitative and Qualitative Evaluation. *Gerontology* **2020**, *66*, 295–303, http://doi.org/10.1159/000504883.
16. Zaslavsky, O.; Thompson, H.J.; McCurry, S.M.; Landis, C.A.; Kitsiou, S.; Ward, T.M.; Heitkemper, M.M.; Demiris, G. Use of a Wearable Technology and Motivational Interviews to Improve Sleep in Older Adults With Osteoarthritis and Sleep Disturbance: A Pilot Study. *Res. Gerontol. Nurs.* **2019**, *12*, 167–173, https://doi.org/10.3928/19404921-20190319-02.
